# Causal attributions of failure among post graduate medical residents in exit fellowship examination in Pakistan: A qualitative study

**DOI:** 10.12669/pjms.39.4.7693

**Published:** 2023

**Authors:** Lamees Mahmood Malik, Tanzeela Khalid, Abid Ashar

**Affiliations:** 1 Prof. Dr. Lamees Mahmood Malik, MBBS, FCPS (Derm) Professor of Dermatology Unit-I, Allama Iqbal Medical College / Jinnah Hospital Lahore, Pakistan; 2 Prof. Dr. Tanzeela Khalid, MBBS, FCPS (Derm), MCPS-HPE. Professor of Dermatology, University Medical & Dental College, The University of Faisalabad, Pakistan; 3 Prof. Dr. Abid Ashar, BDS, FPSRCS (England), MCPS-HPE. Principal, Professor of Oral & Maxillofacial Surgery, Fatima Memorial Hospital College of Dentistry, Lahore, Pakistan. College of Medicine & Dentistry, Lahore, Pakistan

**Keywords:** Academic failure, Academic success, Causal attributions, Educational measurement, FCPS Training, Post graduate Residents

## Abstract

**Background and Objective:**

Causal attributions are reasons given to certain events in life including failure and success. The objective of this qualitative study was to explore the attributions perceived as the cause of failure by post graduate residents, failing the final clinical exit examination of Fellowship of College of Physicians and Surgeons Pakistan (FCPS).

**Methods:**

This exploratory study was conducted from July 2021 to July 2022, at Jinnah Hospital, Lahore. Study population was selected by purposeful maximal variation sampling. A total of ten post graduate residents from four specialties, failing in FCPS part two clinical examination were included. After written informed consent, semi structured face to face, in depth interviews were conducted. Data saturation was achieved after eight interviews after which two more interviews were carried out. All interviews were recorded and transcribed verbatim. Thematic analysis of the data was done by synthesizing codes into categories and themes.

**Results:**

A total of 17 attributions noted were grouped into five categories namely ‘Inadequate exam preparation’, ‘Personal factors,’ ‘Exam related factors,’ ‘Training related factors and ‘Luck’. Two main themes of External and Internal factors emerged from these. Common attributions were; lack of effort (8/10), inadequate knowledge (8/10), family commitments (7/10), luck (8/10) and examiner’s attitude (5/10).

**Conclusions:**

Most of the residents attributed internal, unstable and controllable factors like inadequate knowledge and lack of effort. In addition, external uncontrollable factors of bad luck and harsh attitude of examiners were considered as contributory factors towards failure.

## INTRODUCTION

Causal attributions are reasons given to certain events in life including failure and success.^1^ Attributions are measured across three dimensions, namely locus (external or internal), stability (stable or unstable) and controllability (controllable or uncontrollable).^2^ The reasons individuals allocate to their failure and success, determines how they perceive their performance and may predict how they may behave in future performances.^3^

Attribution theory was first proposed by Fritz Heider in 1958 who identified three major causes including ability, effort and task difficulty as causal attributions.^3^ Julian Rotter in 1966 proposed the theory of locus of control.^3^ The greatest contribution was however by Bernard Weiner who laid the foundations of the present day attribution theory of achievement.^4^ Weiner described that success and failure in academic achievement is attributed commonly to four factors: ability, effort, task difficulty, and luck.^3^ Weiner also described the association of causal thinking and feelings or emotions elicited in failure situations, including guilt, shame, anger and hopelessness etc.^4^

Various researchers have examined casual attributions for success and failure in various academic contexts. In 2018, Taskiran and Aydin reported that the majority of study participants in a foreign language course, attributed academic success to effort, teacher’s motivation and class participation.^5^ Other studies showed that high achiever students significantly attributed internally while low achievers attributed externally towards their success and failure.^6^,^7^

Post graduate medical education is a highly competitive field with a high failure rate. In Fellowship of College of Physicians and Surgeons Pakistan (FCPS) examination, the failure rates are generally higher than the comparable contemporary examinations of the Royal Colleges.^7^ Khan HZ, in 2020 exploring the factors leading to early success highlighted the importance of self-regulation and internal motivation as strategies for success in the FCPS exit examination.^8^ However, there is dearth of literature on causal attributions of failure in post graduate medical residents. The purpose of this study was to explore the patterns of attributions in post graduate medical students regarding failure in exams. As these attributions generally determine how the individual will perform in subsequent examinations, identifying them will guide trainers for attributional retraining. This will not only help them achieve a better outcome next time but will also guide examiners and trainers to rectify the problems identified by participants regarding exam process to make it as fair, unbiased and candidate friendly as possible.

## METHODS

This exploratory study was conducted after approval from the Ethical Review Board Allama Iqbal Medical College (Ref. No.: 57/ERB dated 27-04-2020), from July 2020 to July 2022. Participants included FCPS Part two candidates who failed their clinical examinations. Purposeful maximum variation sampling was used considering gender, marital status, clinical specialty (Medicine, Surgery, Gynecology and Pediatrics), residential status (Boarders or Non-boarders) and one or more attempts to pass the exams. Dermatology candidates were excluded as chief investigator is an examiner for the specialty.

Semi-structured, face to face in depth interviews (with pre-phrased questions) were conducted by the first author after obtaining written informed consent. Participants were briefed about the purpose and nature of the study as well as assured about the anonymity and confidentiality of the data. Data saturation was achieved after eight interviews. Two more interviews were conducted for further confirmation.

The following two questions were asked in this sequence.

1. In your opinion what led to your failure in the FCPS Part Two Exam?
2. What could you have done to have a more favorable result?

Responses were explored in depth based on their answers with probes covering the three dimensions of attribution i.e., locus, controllability and stability of the factors. These included factors at home or hostel, role of luck, gender and examiners bias. Students were specifically asked if they thought they had necessary ability to pass the exam and whether they were able to put in maximum possible effort for the exam.

All interviews were audio recorded and field notes were taken. Verbatim transcriptions of all interviews were done. Interviewees were given the opportunity to delete any comments they wanted at the end of each interview. Member checking of the interviews was done by sending transcriptions for comments about accuracy to interviewees for data validation. Manual coding of the transcripts was done by chief investigator and reviewed by co-authors to reach a consensus code list.

Thematic analysis of the data was done by Miles & Huberman interactive model of data condensation, display and drawing conclusions.^9^ Weiner’s three-dimensional attribution theory model was used as a theoretical framework to synthesize codes into categories and themes.^4^ Description of the experience through verbatim examples was done for each category.

## RESULTS

Seven female and three male residents from Medicine, Surgery, Pediatrics and Gynecology were interviewed. Seven of them were married while three were single. Six were day scholars while four lived in hostels. Four participants had encountered failure for the first time in their life.

A total of 17 attributions were noted. These were grouped into five categories after interpretation and comprehension of trends labelled as ‘Inadequate exam preparation’, ‘Personal factors,’ ‘Exam related factors,’ ‘Training related factors and ‘Luck’. Two main themes of External and Internal factors emerged, which were further aligned with respect to the stability and controllability of these factors. (Table-I).

**Table-I:** Categories Codes and Selected comments.

Themes	Categories	Stable (S) or Unstable (US)	Controllable (C) or Uncontrollable (UC)	Codes (Frequency out of 10)	Selected Comments
Internal factors	Inadequate exam preparation	US	C	Weak theoretical background (8/10)	“I did not have full grip on theory and took a longer time to understand and answer the examiner”
		US	C	Inadequate clinical case practice (3/10)	“I had read theory but my clinical was weak. I had not practiced enough.”
		US	C	Lack of effort (8/10)	“No, it was not my best effort.” “I did make my full effort till that time, but now that I have more time, I will make more effort”
		US	C	Lack of experience of exam format (1/10)	“Exam format was unfamiliar for me I got confused.”
	Personal factors	S	C	Family commitments (7/10)	“I had to look after my babies and other family commitments.” “I even had to attend family weddings close to the exam.” “My family were pressurizing me to get engaged before the exam.”
		US	UC	Medical and health issues (1/10)	“My health issues were the main reason.”
		US	C	Stress (5/10)	“Repeated failures had made me stressed.”
		US	C	Poor confidence (4/10)	“Seeing an examiner known for being strict on my case made me further stressed and unconfident.”
		US	C	Poor time management (3/10)	“I could not finish my long case 2-3 minutes before as everyone else.”
External factors	Exam related factors	US	C	Perception of unfairness (2/10)	“Exam was not uniformly fair. Some who got easy cases or lenient examiners passed easily despite having lesser knowledge.”
		S	C	Examiner’s harsh attitude or inattentiveness (5/10)	“I forgot to thank the patient in the end and my examiner made me realize my mistake in a very harsh way by saying to him very loudly Shukria Buhat Buhat Aap Ka.” “When I was answering he kept on interrupting and didn’t listen to my answer!” “He didn’t evaluate me properly. He was not paying attention.”
		US	UC	Poor facilities in exam venue (2/10)	“It was month of June and there were not even fans in the waiting area”.
	Training related factors	US	C	Inadequate clinical training (1/10)	“My training was deficient. I had not presented enough cases to my supervisor and seniors”
		US	C	-Shortage of time for self-study (3/10)	“We got very little time for preparation. I couldn’t revise properly”
		US	C	Tough duties (2/10)	“Our duties were very tough. I hardly got time to study anything during training.”
		US	C	Slow reflexes due to gap in COVID (1/10)	“We were coming for exam right after COVID and our reflexes were not that sharp.”
	Luck	US	UC	Bad luck (8/10)	“It all depended on luck. I could have been on that side of the room where there were easier cases.” “Yes, luck also had its role but there were other factors too”. “Yes, luck is definitely there but I think luck also favours those who have worked hard”

None of the participants thought that their gender had any role in their failure. Also, none of the interviewees attributed their failure to lack of ability to pass the exam. All participants encountering repeated failures attributed bad luck while only two out of four first-time failures did so. Almost all believed that majority of the factors responsible were unstable and they will be able to overcome them in the subsequent examinations. All interviews ended on a positive note with the participants saying that they will increase their effort to get through the next examination, (Table-II).

**Table-II:** Participants responses to the question: ‘What could you have done to get a more favourable result?’

No	Participants Response	External (E)/ internal (I)	Controllable (C)/ Uncontrollable (UC)	Stable (S) / Unstable (US)
1	I would try to stay calm and cool and have a grasp over things that were deficient	I	C	US
2	Improve my theory and present cases again and again in front of seniors	I	C	US
3	I will improve myself practically by more practice and also improve theory	I	C	US
4	I could have studied more, increase my effort and input, reduce my stress level	I	C	US
5	I should have kept control on my nerves, I shouldn’t have thought I am going to fail after getting a particular examiner	I	C	US
6	I could improve my history and examination skills and practice time management	I	C	US
7	I could have taken more practice sessions with senior teachers	I	C	US
8	I should have had more practice sessions with my colleagues and supervisor and other examiners	I	C	US
9	I need to focus on clinical exam, clinical methods and history	I	C	US
10	I need to focus more on theory, face more examiners, increase my effort and practice	I	C	US

## DISCUSSION

“Success is going from failure, to failure without losing your enthusiasm,” famously quoted by Sir Winston Churchill, fits well with eventual success in exit post-graduate examinations. This research was conducted in this background. Most of the failing participants in our study identified internal controllable factors as being the main reason of their failure. However, a majority also blamed additional external factors to have contributed. (Table-I). Previous studies have shown that high achievers mostly attribute their success and failures to internal, whereas low achievers generally assign external causes to both.^10^,^11^ In a recent study in 2017, failing undergraduate medical students attributed their failures to internal controllable factors which is similar to our study.^12^ Generally medical students, both undergraduate and post graduate are high achievers in their lives, hence their attributions were in line with those of high achieving students.^8^

‘Inadequate exam preparation’ was the foremost reason for failure in our study (10/10). When first seen, ‘lack of study’ and ‘lack of practice’ may appear synonymous with ‘effort’. However, the term ‘effort’ might have different implications for different individuals. Elliott et al. defined effort as ‘a construct with both cognitive and behavioral components.’^13^ Majority of participants in our study (8/10) mentioned lack of effort as a causal attribution. However, a few of them thought that despite making maximum effort they failed due to inadequate learning strategies. The factors identified by them included stress and time management, focusing on weak areas and seeking help and feedback from colleagues and seniors. They believed that with the same effort and an appropriate learning strategy, they could have achieved better. Previous studies have also highlighted the importance of adopting correct learning strategies for achieving success.^8^

Personal factors included family commitments and stress in seven and five out of 10 participants respectively. The causes of stress identified included repeated failures, facing an examiner known to be strict and unfamiliarity with the exam format. Other researchers have also highlighted that personal problems faced by medical students can adversely affect academic results.^8^,^14^ A higher occurrence of stress, anxiety and depression may lead to sleep disturbance and interferes with preparation, concentration and performance.^15^,^16^ The added responsibilities of supporting and caring for a family can increase stress and frustration.^8^ Stress was found to be a common factor in both low and high achievers. While in low achievers it was associated with fear in high achievers it acted as a driving force.^17^

Examiners’ attitude was mentioned by half of our study participants (5/10) as a contributory factor. Although not outrightly biased against them, they had the impression that the examiners they faced were harsh, inattentive, sarcastic, and generally unfriendly and this stress contributed to their poor performance. Studies have shown that the more experienced examiners tend to be stricter while junior ones show more leniency.^18^ Clinical experience or seniority of the examiners does not necessarily mean consistency and fairness of the exam.^19^ Examination itself is a stressful event and examiner’s attitude adding to it may be perceived by the candidate as an attribution.^20^ Examiners in clinical exams are subject to many forms of bias e.g. mood of the examiner, familiarity with the candidate and forming an overall first impression, which may affect judgement and contribute to the overall score of the candidate. In high stakes examinations, this can have serious consequences for not only the candidate but also for the medical profession.^21^ Training of examiners can lead to better standardization of exam scoring.^22^

Training related factors, including shortage of time for preparation, tough duties, and deficient training were also held responsible by our study participants. Khan HZ et al observed that the challenges faced by the residents were related to increased workload, thus compromising opportunities for training.^8^ Simpkin et al. also observed that cognitive capabilities of the residents deteriorated due to longer duty hours.^23^

Luck was attributed by a substantial majority of our participants to be part of the reason of their failure. However, they also believed that luck was not the only factor responsible for their failure (Table-I). Luck as an attribution is perceived by many students to justify high or low performance.^24^ Farid and Akhtar have reported that the majority of low achievers, attributed their failure to bad luck.^11^ It has also been observed that people from different cultural and ethnic backgrounds may give more importance to luck than others.^4^

The four participants who had failed for the first time ascribed unfamiliarity of the exam format, stress, and inadequate preparation as their main attributions. While only half of the first-time failures attributed luck, all encountering repeated failures thought it had some role in their failure. Similarly examiner’s attitude was attributed less by first time failures (1/4) as compared to repeated failures (4/6). This indicates that generally candidates tend to attribute externally as they encounter repeated failures. Gender differences in attributions have also been reported in literature.^24^,^25^ However these were not highlighted in our study participants probably because majority of them were females (7/10).

This study explored the patterns of attributions in post graduate medical residents, regarding failure in exit clinical exams. The attributions of failing post graduate trainees can provide useful information to help improve the examination system as well as the clinical training. It can also highlight areas where attributional retraining can help them come up with a successful outcome.

### Limitations:

The study included residents of only four specialties of FCPS training programs from one institution only. The results and conclusions should accordingly be interpreted for transferability.

## CONCLUSIONS

The results of this study conclude that most of the post graduate FCPS trainees believed that they can themselves control their examination fate. Majority attributed internal, unstable and controllable factors as being the main cause of their failure. External factors including bad luck, personal commitments and harsh attitude of examiners also played a contributory role.

### Authors’ Contribution:

**LMM:** Conceptualization, data collection, statistical analysis, writing & editing.

**TK & AA:** Conceptualization, data analysis, writing, critical revision and editing of manuscript.

All authors are responsible and accountable for the accuracy of the work.
